# Effect of the type of reducing agents of silver ions in interpolyelectrolyte-metal complexes on the structure, morphology and properties of silver-containing nanocomposites

**DOI:** 10.1038/s41598-020-64079-0

**Published:** 2020-04-28

**Authors:** V. Demchenko, S. Riabov, S. Kobylinskyi, L. Goncharenko, N. Rybalchenko, A. Kruk, O. Moskalenko, M. Shut

**Affiliations:** 1 Institute of Macromolecular Chemistry, the National Academy of Sciences of Ukraine, Kyiv, Ukraine; 2 Zabolotny Institute of Microbiology and Virology, the National Academy of Sciences of Ukraine, Kyiv, Ukraine; 3 Nizhyn Gogol State University, Nizhyn, Ukraine; 4 Dragomanov National Pedagogical University, Kyiv, Ukraine

**Keywords:** Chemistry, Polymer chemistry, Nanocomposites

## Abstract

The objective of this work is to study the peculiarities of structural organization, morphology, thermomechanical, electrical and antimicrobial properties of nanocomposites based on pectin-polyethyleneimine interpolyelectrolyte complexes and silver nanoparticles in dependence on the type of reducing agent being applied for chemical reduction of silver ions in the interpolyelectrolyte-metal complexes. The average size of Ag nanoparticles is shown to be increased with decreasing of the activity of reducing agent (*E*_0_) and equals to 3.8 nm, 4.3 nm, and 15.8 nm, respectively, when engaging sodium borohydride (–1.24 V), hydrazine (–1.15 V) and ascorbic acid (–0.35 V). Moreover, it was found that the crystallite size of Ag nanoparticles also had the smallest value for nanocomposites obtained involving NaBH_4_ as reducing agent. Ag-containing nanocomposites prepared by reduction of silver ions in interpolyelectrolyte-metal complexes while applying a range of reducing agents are characterized by different electrical properties and polymer matrix’ glass transition temperature. The influence of silver nanoparticles’ size incorporated in the polymer matrix on the antimicrobial activity of nanocomposites has been established. The inhibition zone diameter of *Staphylococcus aureus* and *Escherichia coli* was higher for nanocomposites obtained using sodium borohydride and hydrazine compared to nanocomposites where ascorbic acid was used as the reducing agent.

## Introduction

Within the last decades, interest in studying of nanodimensional particles of various metals is constantly grown^1,2^. First of all, it is explained by their unique optical, electronic, catalytic and other properties, which sharply distinguish them from their analogs – microscale objects. Therefore, the hybrid materials containing silver nanoparticles are perspective for development of catalytic systems and also for use in optoelectronics and nanophotonics^3,4^. Furthermore, nanocomposite materials with silver nanoparticles are widely applied as effective antibacterial and antiviral medicines^5–10^.

In particular, for Ag-based nanocomposites’ formation authors^11^ used the following reducing agents, like ascorbic acid, sodium borohydride, hydrazine, sodium citrate, glucose and polyvinyl alcohol as capping agent.

Particle size of Ag being formed after reduction was smaller and dependent on the reduction potential of the reducing agents. In contrary, in^12^ authors shown that average particles size of Ag formed, when adding Tween-20 as stabilizer, was 50 nm and 80 nm, implying sodium borohydride and hydrazine as reducing agents, respectively. In general^13^, average particle size of Ag turned out to be smaller when applying NaBH_4_, but it depends on various factors, namely, molar ratio of silver to a reducing agent and capping agent, pH, temperature etc.

Crucial problem to be solved, while forming metal-containing polymer nanocomposites is to achieve the desired shape, size, and uniform distribution of nanoparticles in the polymer matrix. Interpolyelectrolyte complexes allow to stabilize nanoparticles in the polymer matrix, protecting them from aggregation processes^4^.

So, the aim of this work is to study the features of the structural organization, thermomechanical, electrical and antimicrobial properties of nanocomposites based on interpolyelectrolyte complexes comprising natural and synthetic components (pectin–polyethylenimine) as well as the Ag nanoparticles fabricated by chemical reduction of silver ions with different reducing agent.

## Experimental

### Materials

To obtain the interpolyelectrolyte complexes (IPEC), pectin–polyethyleneimine; the interpolyelectrolyte–metal complexes (IMC), pectin–Ag^+^–polyethyleneimine; and nanocomposites of IPEC–Ag the following reagents were used: anionic polyelectrolyte citrus pectin (Cargill Deutschland GmbH, Germany) with *М*_n_ = 3 × 10^4^, cationic polyelectrolyte anhydrous branched polyethyleneimine (PEI) (Aldrich) with *М*_n_ = 1 × 10^4^ and *М*_w_ = 2.5 × 10^4^ g/mol, silver (I) nitrate (AgNO_3_) (Aldrich), sodium borohydride (NaBH_4_) (Aldrich), hydrazine (N_2_H_4_·H_2_O) (Merck), ascorbic acid (C_6_H_8_O_6_) (Aldrich)^10^.

### Preparation of polymer systems

IPEC samples were formed via mixing of 5% aqueous solutions of pectin and PEI taken at a molar ratio of 1:1, at *Т* = 20 ± 2 °С. IPEC as films were prepared via pouring their solutions onto polytetrafluoroethylene (PTFE) plates and then dried at *Т* = 20 ± 2 °С up to constant weight. Dried (water insoluble) IPEC films were washed from unreacted components of oppositely charged polyelectrolytes in distilled water up to neutral pH and dried repeatedly at 20 ± 2 °С up to constant weight. The resulting films were 100 μm of thickness. IMC samples were prepared via immersion of IPEC films into an aqueous solution of AgNO_3_ with a concentration of 0.1 mol/L at *Т* = 20 ± 2 °С for 24 h. The colorless IPEC films became dark red. The sorption capacities of films, *А* (mmol/g), were calculated through the formula

$$A=({c}_{init}\,-\,{c}_{eq})V/m,$$

where *m* is the weight of the sorbent, *V* is the volume of silver nitrate’s solution, and *c*_init_ and *c*_eq_ are the initial and the equilibrium concentrations of silver ions. For IMC films *А* = 5.0 mmol/g. Chemical reduction of Ag^+^ ions in the volume of IMC was carried out by means of such reducing agents: sodium borohydride (NaBH_4_), hydrazine (N_2_H_4_) and ascorbic acid (C_6_H_8_O_6_). Reduction of silver ions with NaBH_4_ was performed (the molar ratio [BH_4_^−^]: [Ag^+^] = 3.0) in alkaline medium at pH 10.8 in aqueous solution during 3 h at *T* = 20 ± 2 °C (until gas evolution ceased). Reduction of silver ions by means of N_2_H_4_ (molar ratio [N_2_H_4_]:[Ag^+^] = 3.0) was carried out in alkaline medium at pH 13 ([NaOH]:[N_2_H_4_] = 1.0) in water solution during 3 h at *T* = 60 ± 2 °C. Reduction of silver ions with ascorbic acid was realized (the MC [C_6_H_8_O_6_]:[Ag^+^] = 3.0) in aqueous solution at pH 2.7 during 3 h at *T* = 60 ± 2 °C. Concentration of reducing agents in solutions was 0.1 mol/l. As a result of the reduction the colour of films changes from red to silvery. After that all samples were washed with alcohol and dried at ambient temperature up to the constant weight^10^.

### Experimental methods

The features of the structural formation of the IPEC (pectin–PEI); the IMC (pectin–Ag^+^–PEI); and nanocomposites of IPEC–Ag were studied by wide-angle X-ray diffraction on a DRON-4–07 diffractometer, whose X-ray optical scheme was used to “pass” primary-beam radiation through samples. X-ray diffraction studies were performed at *Т* = 20 ± 2 °С in Cu*К*_α_ radiation monochromated with a Ni filter. The size of the Ag nanoparticles and their distribution in the polymer matrix were examined with a JEM-1230 transmission electron microscope (JEOL, Japan) at a resolution of 0.2 nm^2.^ Thermomechanical studies of polymer systems were conducted using the penetration method in the mode of a uniaxial constant load (σ = 0.5 MPa) on a UIP-70M device. Linear heating of samples was performed at a rate of 2.5 °С/min in the temperature range −100 to +350 °С. The frequency dependence of the real part of the complex ac conductivity of a polymer system, σ_ac_(*f*), was determined with the use of dielectric spectroscopy implemented with an R5083 ac bridge;^14^ σ_ac_(*f*) was estimated from the relationship σ_ac_(*f*) = 2π*f*ε′(*f*)tanδε_0_, where *f* is the frequency, ε′(*f*) = *С*(*f*)/*С*_0_ is the frequency dependence of the polymer permittivity, *С* is capacity of the measuring capacitor with a sample, *С*_0_ is capacity of a capacitor filled with air, and ε_0_ = 8.85·10^−12^ F/m is the electric constant. Measurements were performed in the frequency range 10^2^–10^5^ Hz and temperature range of 20–100 °C. The antimicrobial activity of IPEC–Ag nanocomposites, prepared by chemical reduction of Ag^+^ ions in IMC was investigated using reference strains of opportunistic bacteria *Staphylococcus aureus* ATCC 6538 and *Escherichia coli* ATCC 35218 (as a model gram-positive and gram-negative bacteriа)^1^.

## Results and Discussion

### Peculiarities of structure and morphology of silver-containing nanocomposites

**Figure 1:**
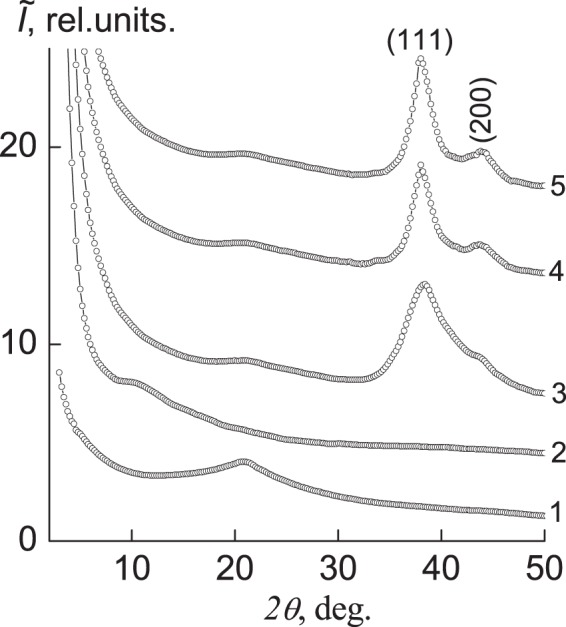
Wide-angle X-ray diffractograms of the (**1**) IPEC, (**2**) IMC and silver-containing nanocomposites obtained by chemical reduction of silver ions in IMC with (**3**) sodium borohydride, (**4)** hydrazine, (**5**) ascorbic acid.

The analysis of wide-angle X-ray patterns (Fig. 1, curve 1) has shown that stoichiometric IPEC formed by the equimolar quantity of anionic and cationic polyelectrolytes, namely pectin and PEI, is characterized by short-range ordering during translation of fragments of oppositely charged polyelectrolytes’ macromolecular chains in space.

This indicates the appearance of one diffuse diffraction maximum at 2*θ*_*m*_ ≈ 20.8° on the X-ray patterns of the IPEC. The average value of the period of short-range ordering of fragments of complementary macromolecular chains of oppositely charged polyelectrolytes in the IPEC (the Bragg distance between the layers of macromolecule chains) according to the Bragg equation is:

$$d=\lambda {(2{\rm{s}}{\rm{i}}{\rm{n}}{\theta }_{m})}^{-1},$$

where the *λ* is the wavelength of characteristic X-ray radiation (*λ* = 1.54 Å for the Cu*K*_*α*_ radiations), equals 4.3 Å.

Sorption of AgNO_3_ by IPEC and then formation of the interpolyelectrolyte metal complexes (pectin–Ag^+^–PEI) results in changing of diffraction picture. So, on diffractograms the intensive diffusion diffraction maximum at 2*θ*_*m*_ ~ 11.2° is emerged, characterizing the structure of IMC (curve 2)^10^. At the same time, amorphous halo related to the IPEC’s structure disappears at 2*θ*_*m*_ ~ 20.8°. It points out to full transformation of interpolyelectrolyte complexes into interpolyelectrolyte metal complexes in the course of adsorption of silver ions.

Chemical reduction of Ag^+^ ions in interpolyelectrolyte-metal complexes with the usage of sodium borohydride at molar ratio [BH_4_^−^]:[Ag^+^] = 3.0, which is determined to be optimal^10^, leads to formation of nanocomposite based on IPEC and Ag nanoparticles.

Thus, diffraction maximum specific to interpolyelectrolyte-metal complexes’ structure is absent at 2*θ*_*m*_ ~ 11.2° (Fig. 1, curve 3), but two intensive maxima at 2*θ*_*m*_ = 38.2° and 43.8°, corresponding to the of silver face-centered cubic lattice’s crystallographic planes with Miller indices (111) and (200), respectively, confirm the presence of metal silver in the system.

The assessment of an average crystallite size of Ag nanoparticles in IPEC was carried out by the Scherrer method^1^:

$$L=K\lambda {(\beta \cos {\theta }_{m})}^{-1},$$

where K a constant, connected with a form of crystallites (when unknown form K = 0.9), and *β* is the full-width at half maximum of a singlet diffraction maximum of discrete type. It was shown that for the silver-containing nanocomposites obtained by reduction of silver ions by sodium borohydride *L* ≈ 2.7 nm (Fig. 1, curve 3). Silver-containing nanocomposites (pectin-Ag-PEI) obtained by chemical reduction of silver ions by means of both hydrazine and ascorbic acid, have a bit different structure. In particular, the crystallites’ average size of silver nanoparticles in these nanocomposites is *L* ≈ 3.2 nm (curves *4–5*). For calculations, the diffraction maxima at 2*θ*_*m*_ = 38.2° and 43.8° were used (curves *3–5*).

**Figure 2:**
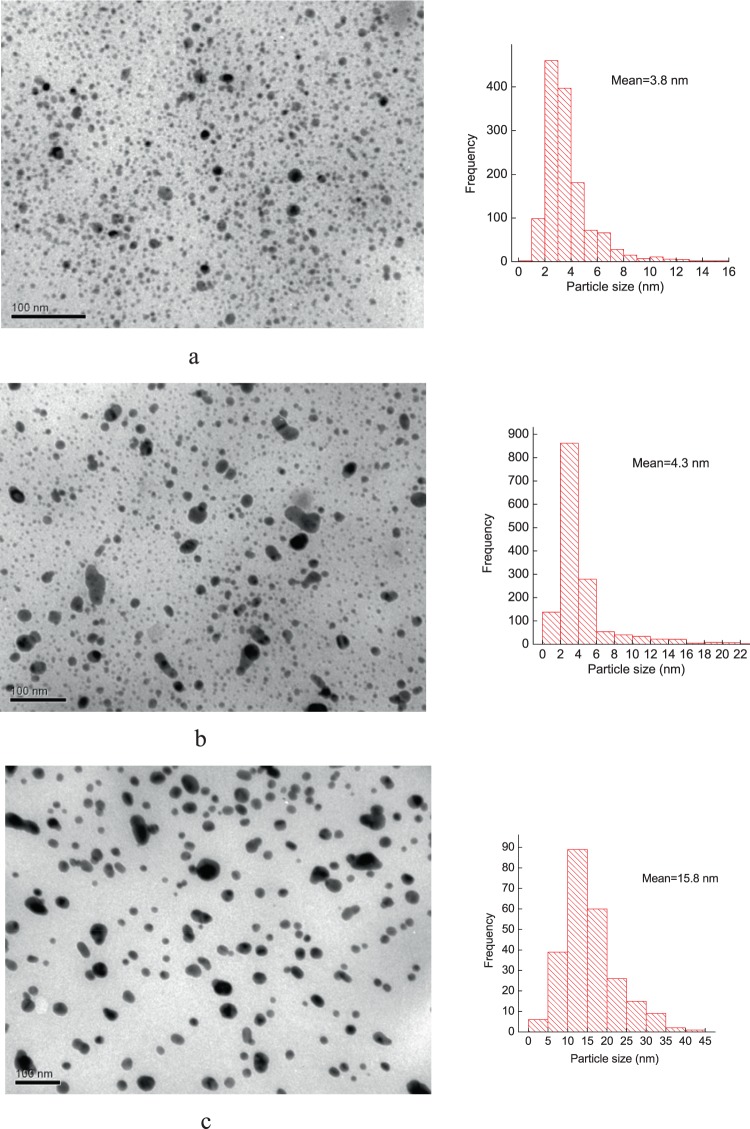
TEM images of the silver-containing nanocomposites produced by using various reducing agents: (**a**) sodium borohydride; (**b**) hydrazine; (**c**) ascorbic acid.

The analysis of TEM images of the silver-containing nanocomposites prepared by chemical reduction of silver ions, involving various reducing agent revealed that nanoparticles of smallest size are formed, when reduction of silver ions by sodium borohydride (3.8 nm) takes place, and with biggest size – when the ascorbic acid being used (15.8 nm) (Fig. 2). At the same time the narrowest size distribution of Ag nanoparticles in polymeric matrices is observed when hydrazine to be applied. Thus, our results indicates that decreasing in redox potentials of reducing agent leads to increasing of nanoparticles size.

A detailed distribution of nanoparticles by size is presented in Table 1.

**Table 1:** Distribution of nanoparticles by size.

IPEC-Ag (NaBH_4_)	**The size of nanoparticles, nm**									
	0–1	1–2	2–3	3–4	4–5	5–6	6–7	7–8	9–10	>10
	**Content of nanoparticles by size, %**									
	0.15	7.3	33.9	29.3	13.3	5.3	4.9	2.1	1.1	2.65
IPEC-Ag (N_2_H_4_)	**The size of nanoparticles, nm**									
	0–2	2–4	4–6	6–8	8–10					>10
	**Content of nanoparticles by size, %**									
	5.4	51.2	27	10.2	2.9					3.3
IPEC-Ag (C_6_H_8_O_6_)	**The size of nanoparticles, nm**									
	0–5	5–10	10–15	15–20	20–25	25–30				>30
	**Content of nanoparticles by size, %**									
	2.4	15.8	36	24.3	10.5	6.1				4.9

### Thermomechanical behavior of the polymer system

**Figure 3:**
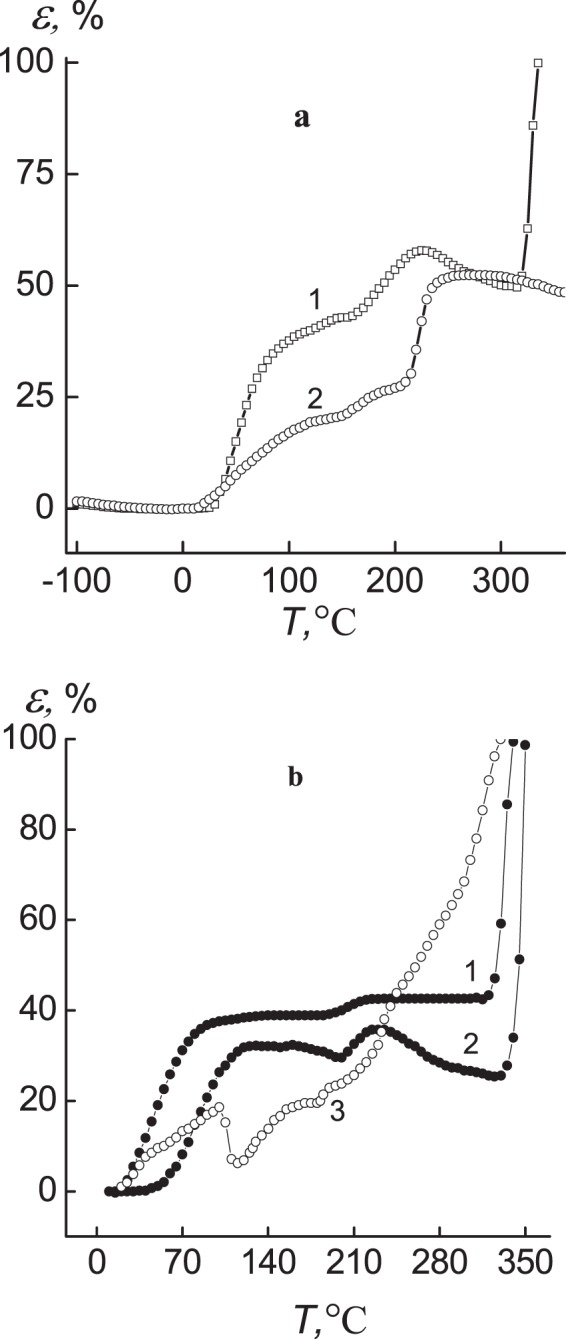
Thermomechanical curves a: (**1**) IPEC, (**2**) pectin and b: the silver-containing nanocomposites obtained by using the (1) sodium borohydride, (2) hydrazine, (3) ascorbic acid.

Taking into account the structural and morphological features and specific behavior of silver-containing nanocomposites, it was also important to investigate their thermomechanical properties. The analysis of a thermomechanical curve of IPEC (Fig. 3a, curve 1) shows that temperature transitions intrinsic to a glass transition temperature and a viscous-flow temperature are in the ranges of 25–145 °C and 265–335 °C, respectively. Furthermore, in the range from 150 to 245 °C temperature transition caused by melting of pectin crystallites in IPEC (curves *1, 2*) is seen^2^.

Thermomechanical behavior of silver-containing nanocomposites depending on the type of reducing agent is found to be different (Fig. 3b). First of all, it concerns the values of glass transition temperature that is unexpected high for sample obtained with N_2_H_4_. Whereas, the others composites had a lower values, which is characteristically for such systems, because nanoparticles disrupt hydrogen bonds and thus increase mobility of polymer chains. The plausible explanation for the high value of *T*_g_ may be due to cross-linking of polymer system, which leads to decrease of chains mobility. Temperature transition in the area 195–245 °C for nanocomposites developed with NaBH_4_ and N_2_H_4_ is attributed to melting of pectin crystallites in the IPEC (curves *1, 2*)^2^.

Thermomechanical curve of the sample obtained with ascorbic acid, unlike others, has several transition temperatures: 44, 100, 115 and 180 °C (curves 3). The transitions at temperatures 44, 100 and 115 °C could be assigned to destruction, crystallization and melting of composite part that is formed as a result of the attachment of excess of ascorbic acid to amino group of PEI. In its turn, temperature transition at 180 °C is connected with melting of pectin part in composite. The values of temperature transitions of the studied polymeric systems are given in Table 2.

**Table 2:** Glass transition temperature of silver-containing nanocomposites.

The polymeric systems obtained by using various reducing agent	*Т*_*g*_,	*Т*_*f*_,
	°С	°С
Pectin	60	–
IPEC	53	319
IPEC–Ag (NaBH_4_)	50	323
IPEC–Ag (N_2_H_4_)	82	335
IPEC–Ag (C_6_H_8_O_6_)	61	210

### Electrical properties of the polymer system

**Figure 4:**
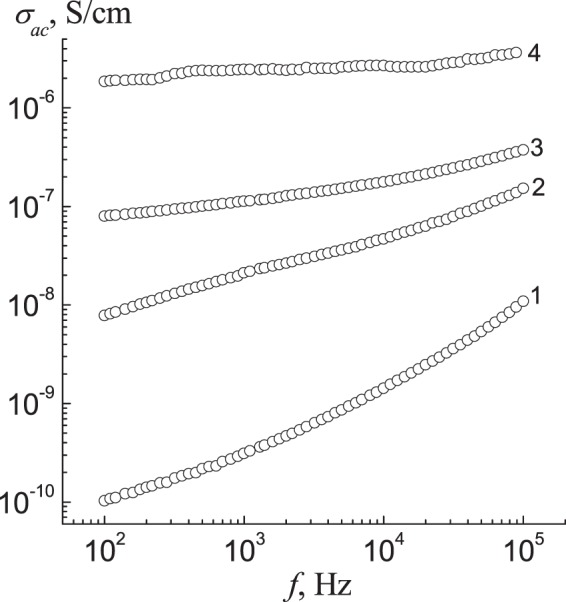
Frequency dependences of the real part of AC conductivity for IPEC (**1**) and IPEC–Ag which obtained by using N_2_H_4_ (**2**), ascorbic acid (**3**) and NaBH_4_ (**4**) at *T* = 20 ± 2 °C.

The study of the frequency dependence of the real part of complex conductivity, σ_ac_(f), showed that IPEC exhibits dielectric properties and the IPEC–Ag nanocomposites demonstrate a semiconductor properties (Fig. 4). So, σ_ac_ value is found to be increased by up to 2–4 orders of magnitude at ambient temperature, while transferring from IPEC to IPEC–Ag nanocomposites. At elevated temperature the difference was significantly smaller (approximately one order of magnitude or less). The high value of conductivity at 20 °C was observed for sample obtained with NaBH_4_, but at elevated temperatures (80–100 °C) σ_ac_ was higher for nanocomposite, prepared with ascorbic acid (Table 3). Such behaviour is explained by the difference in glass transition temperature of the samples.

**Table 3:** Conductivity value of IPEC and IPEC–Ag at different temperatures (*f* = 1 kHz).

Samples	σ_ac_, S/cm					
	20 °C	40 °C	50 °C	60 °C	80 °C	100 °C
IPEC	3.12·10^−10^	1.10·10^−9^	1.39·10^−8^	2.56·10^−8^	2.95·10^−7^	1.40·10^−6^
IPEC–Ag (NaBH_4_)	2.74·10^−6^	3.09·10^−6^	3.09·10^−6^	3.17·10^−6^	4.01·10^−6^	6.64·10^−6^
IPEC–Ag (N_2_H_4_)	2.13·10^−8^	9.42·10^−8^	3.47·10^−7^	7.58·10^−7^	2.88·10^−6^	4.95·10^−6^
IPEC–Ag (C_6_H_8_O_6_)	1.13·10^−7^	1.32·10^−6^	2.08·10^−6^	4.91·10^−6^	1.41·10^−5^	3.27·10^−5^

The above data show that the conductivity increases with the temperature and it may be due to enhance in chain mobility.

### Antimicrobial properties of the polymer systems

**Figure 5:**
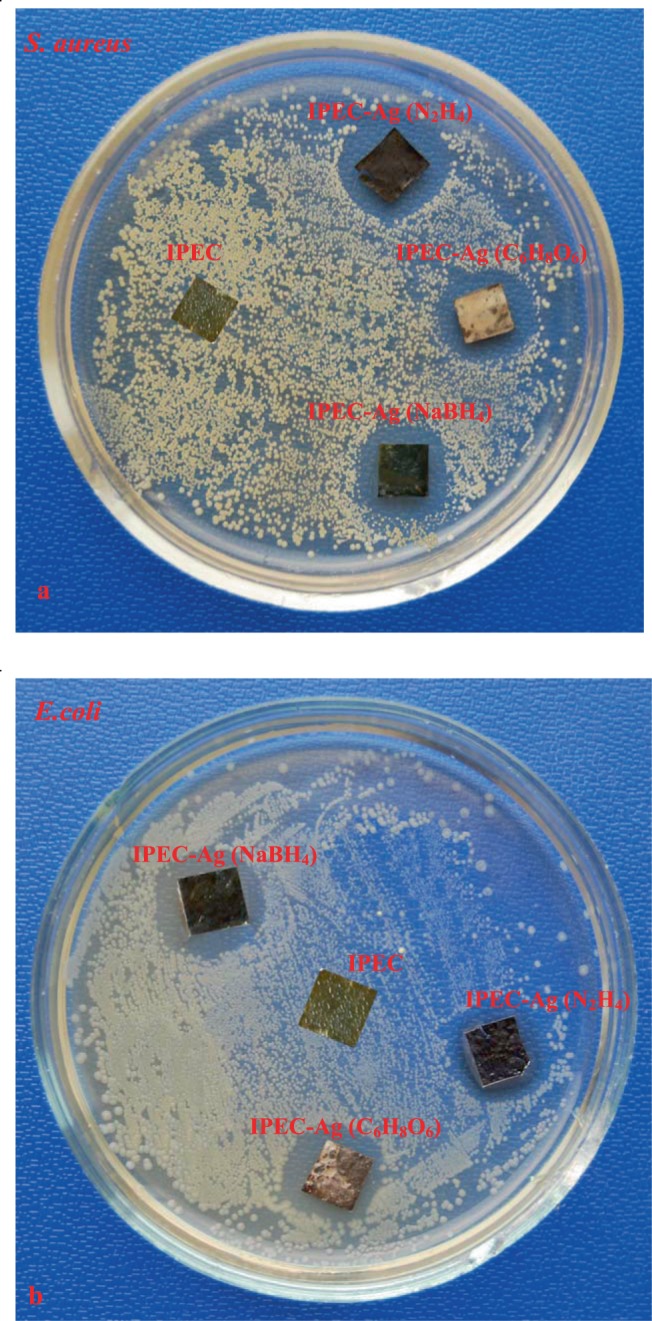
Images of antimicrobial test results of agar plates Ag-containing nanocomposites, obtained via chemical reduction of Ag^+^ ions in the polymer films against *S. aureus* (**a**) and *E. coli* (**b**).

Testing of the antimicrobial properties of the elaborated nanocomposites IPEC–Ag showed they have high antimicrobial activity towards strains of *S. aureus* and *E. coli* (Fig. 5 and Table 4). After incubation during 24 h at 37 °C, it is recorded an accurate zone around contours of film, free from microorganisms that demonstrates inhibition of bacterial growth. In control samples (the polymeric film without nanoparticles) the active growth of the studied bacteria could be seen.

**Table 4:** Antimicrobial activity of nanocomposites IPEC-Ag produced by various reductants.

Reducing agent	Diameter of the inhibition zone, mm	
	*Staphylococcus aureus*	*Escherichia coli*
NaBH_4_	IPEC–Ag	IPEC–Ag
	18,2 ± 0,8	17,6 ± 0,6
N_2_H_4_	IPEC–Ag	IPEC–Ag
	19 ± 0,6	20 ± 0,6
C_6_H_8_O_6_	IPEC–Ag	IPEC–Ag
	17 ± 0,6	16 ± 0,6
control sample	IPEC	IPEC
	0	0

The nanocomposites developed with sodium borohydride and hydrazine have the higher antimicrobial activity in comparison to the nanocomposites where ascorbic acid was used as the reducing agent. It could be elucidated by the size of silver nanoparticles in the polymeric matrices. The antimicrobial activity of the elaborated silver-containing nanocomposites is listed in Table 4.

## Conclusions

Novel silver-containing nanocomposites formed on the base of interpolyelectrolyte complexes (IPEC) with silver incorporated were elaborated and their structural characteristics’ peculiarities and antimicrobal activity were investigated. For preparing those nanocomposites different reducing agents were applied, precisely sodium borohydride, hydrazine, ascorbic acid. So, nanocomposites having crystallites size (*L*) 2.7 nm were established by WAXS method to be formed with reducing agent NaBH_4_, at the same time, involving of N_2_H_4_ and C_6_H_8_O_6_ gives value *L* ≈ 3.2 nm.

TEM demonstrates that Ag-nanoparticles average size are varied, depending on the type of reducing agents – 3.8 nm (NaBH_4_), 4.3 nm (N_2_H_4_), and 15.8 nm (C_6_H_8_O_6_) and it is in good agreement with WAXS data.

Thermomechanical behavior of nanocomposites on the base of ascorbic acid is found to be different compared to other reducing agents. This is probably due to formation of polymer’s part, comprising of an excess of ascorbic acid attached to amino group of PEI.

Investigation of the electric properties of silver-containing nanocomposites showed that IPEC exhibits dielectric properties, meanwhile, the IPEC–Ag nanocomposite, obtained with hydrazine, sodium borohydride, and ascorbic acid demonstrate a semiconductor properties.

Conductivity (σ_ac_) value is found to be enhanced by up to 2–4 orders of magnitude at ambient temperature, while transferring from IPEC to IPEC–Ag nanocomposites. This parameter is corresponding to a type of reducing agent used.

The nanocomposites developed with sodium borohydride and hydrazine have the higher antimicrobial activity in comparison to the nanocomposites where ascorbic acid was used as the reducing agent. It could be elucidated by the size of silver nanoparticles in the polymeric matrices.

## References

[1] Demchenko V (2017). X-ray study of structural formation, thermomechanical and antimicrobial properties of copper-containing polymer nanocomposites obtained by the thermal reduction method. Eur. Polym. J..

[2] Demchenko, V., Shtompel, V. & Riabov, S. Nanocomposites based on interpolyelectrolyte complex and Cu/Cu_2_O core–shell nanoparticles: Structure, thermomechanical and electric properties. *Eur. Polym. J.* **75**, 310–316 (2016).

[3] Pomogailo, A. D. & Kestelman, V. N. Metallopolymer nanocomposites, Springer, New York 564 р (2005).

[4] Zezin AA (2016). Synthesis of Hybrid Materials in Polyelectrolyte Matrixes: Control over Sizes and Spatial Organization of Metallic Nanostructures. Polym. Sci..

[5] Deng Z (2012). Synthesis of PS/Ag Nanocomposite Spheres with Catalytic and Antibacterial Activities. ACS Appl. Mater. Interfaces.

[6] Prozorova GF (2014). Green synthesis of water-soluble nontoxic polymeric nanocomposites containing silver nanoparticles. Int. J. Nanomed..

[7] Barud, H.S. *et al*. Antimicrobial Bacterial Cellulose-Silver Nanoparticles Composite Membranes, *J. Nanomater*, 1–8 **(**2011).

[8] Medhat D (2017). Effect of Au-dextran NPs as anti-tumor agent against EAC and solid tumor in mice by biochemical evaluations and histopathological investigations. Biomed. Pharmacother..

[9] Yan JH, Abdelgawad AM, El-Naggar ME, Rojas OJ (2016). Antibacterial activity of silver nanoparticles synthesized *In-situ* by solution spraying onto cellulose. Carbohydr. Polym..

[10] Demchenko V, Riabov S, Kobylinskyi S, Goncharenko L, Rybalchenko N (2018). Structural Peculiarities and Properties of Silver-Containing Polymer Nanocomposites, Nanochemistry, Biotechnology, Nanomaterials, and Their Applications. Springer Proceedings in Physics, Springer.

[11] Roto R, Rasydta HP, Suratman A, Aprilita NH (2018). Effect of Reducing Agents on Physical and Chemical Properties of Silver Nanoparticles. Indones J. Chem..

[12] Seo W-S, Kim T-H, Sung J-S, Song KC (2004). Synthesis of silver nanoparticles by chemical reduction method. Korean Chem. Eng. Res..

[13] Egorova, E. M., Kubatiev, A. A. & Schvets, V. I. Biological effects of metal nanoparticles, Cham., Springer 292 (2016).

[14] Demchenko VL, Shtompel VI, Riabov SV (2015). DC Field Effect on the Structuring and Thermomechanical and Electric Properties of Nanocomposites Formed from Pectin–Cu^2+^–Polyethyleneimine Ternary Polyelectrolyte–Metal Complexes,. Polym. Sci. A.

